# Evaluating the mediating role of gestational phthalate and replacement biomarkers in the associations between race/ethnicity and birthweight

**DOI:** 10.1097/EE9.0000000000000506

**Published:** 2026-08-07

**Authors:** Amir J. Lueth, Danielle R. Stevens, Sarahna A. Moyd, Elena Sinkovskaya, Ann Przybylska, Antonia M. Calafat, Julianne C. Botelho, George Saade, Alfred Abuhamad, Kelly K. Ferguson

**Affiliations:** a Epidemiology Branch, National Institute of Environmental Health Sciences, Durham, North Carolina; b Division of Epidemiology, Department of Radiology, University of North Carolina at Chapel Hill, Chapel Hill, North Carolina; c Division of Maternal-Fetal Medicine, Department of Obstetrics and Gynecology, Eastern Virginia Medical School, Norfolk, Virginia; d Division of Laboratory Sciences, National Center for Environmental Health, Centers for Disease Control and Prevention, Atlanta, Georgia

**Keywords:** Pregnancy, Endocrine disruptors, Phthalates, Fetal growth, Mediation analysis

## Abstract

**Background:**

Gestational exposure to phthalates is higher among non-Hispanic black (NHB) women than non-Hispanic white (NHW) women and may contribute to racial/ethnic differences in birthweight z-scores.

**Objective:**

We aimed to investigate whether gestational phthalate and replacement biomarkers mediate the association between race/ethnicity and birthweight z-scores.

**Methods:**

We analyzed data from 271 pregnancies in the Human Placenta and Phthalates study, a prospective cohort recruited from 2017 to 2020. Urinary phthalate metabolite concentrations were measured at up to eight time points during pregnancy and averaged to represent gestational exposure. Metabolites were grouped by parent compound. Linear regression models estimated associations in phthalate biomarkers by race and ethnicity, between race and ethnicity and birthweight z-scores, and between phthalate biomarkers and birthweight z-scores. Mediation analyses assessed whether phthalate biomarkers mediate the association between race/ethnicity and birthweight z-scores.

**Results:**

NHB participants exhibited higher concentrations of most phthalate biomarkers than NHW participants. For example, monoethyl phthalate concentrations were 250% higher (95% confidence interval [CI] = 173, 349). NHB participants had 0.43 lower birthweight z-scores (95% CI = −0.67, −0.19) than NHW participants. Monoethyl phthalate and summed metabolites of di-iso-butyl phthalate (ΣDiBP) and di-n-butyl phthalate were inversely associated with birthweight z-scores. ΣDiBP was associated with 0.16 (95% CI = −0.32, −0.002) lower birthweight z-scores. Mediation by phthalate biomarkers was modest and not statistically significant, with a ≤5% mediated effect.

**Conclusions:**

Racial/ethnic differences in phthalate exposure and birthweight z-scores were evident, and some phthalate biomarkers were associated with lower birthweight z-scores. However, phthalate exposures explained only a small, nonsignificant portion of the association between race and ethnicity and birthweight z-scores, underscoring the need to investigate additional contributors.

**What this study adds**

We believe our study illustrates the utility of a mediation analysis framework, which remains underutilized in this field, for evaluating how an environmental exposure may be involved in the relationship between race and ethnicity and adverse pregnancy outcomes.

## Introduction

Phthalates are a class of synthetic nonpersistent endocrine-disrupting chemicals (EDCs) widely used as plasticizers, solvents, and coatings.^1,2^ Due to their widespread use in personal care products, food packaging, common household items, and building materials, phthalates are ubiquitous in the environment.^3,4^ Exposure to phthalates and their replacements is common during pregnancy because they can leach from products and enter the body through inhalation, dermal absorption, and ingestion.^5^ Exposure to phthalates during pregnancy has been associated with perturbations in fetal growth (e.g., lower birthweight).^6–8^

Appropriate fetal growth is a crucial indicator of maternal and infant health outcomes.^9^ Adverse outcomes linked to impaired fetal growth include stillbirth, neonatal morbidity, long-term metabolic disorders (e.g., increased risk of obesity), and childhood cognitive development issues.^9–12^

Differences in fetal growth across racial and ethnic groups have been well-documented^6,13^ and may be influenced by a complex interplay of environmental, systemic, and socioeconomic factors.^6^ Non-Hispanic black (NHB) infants tend to have lower birthweights than non-Hispanic white (NHW) and Hispanic infants.^13–17^ Research suggests that exposure to EDCs such as phthalates can be disproportionately higher in NHB pregnant women compared with others^15,18,19^ and thus may play a role in the fetal growth differences observed by race/ethnicity.^6^

In recent years, there has been an increase in the evaluation of environmental pollutants as potential mediators of the relationships between race and adverse pregnancy outcomes.^20–23^ There is a large body of literature on 1) differences in phthalate exposure by race/ethnicity; 2) differences in birthweight by race/ethnicity; and 3) associations between phthalate exposure and replacement biomarkers and birthweight. However, research investigating the potential role of phthalates, or any environmental exposure, in mediating the relationship between race/ethnicity and fetal growth is limited. To our knowledge, only one study to date has examined the mediating role of EDC exposure in the relationship between race/ethnicity and an adverse pregnancy outcome. In that analysis, polybrominated diphenyl ethers accounted for 11.8% of the total NHB versus NHW differences in gestational age at delivery.^24^

In this study, we aimed to apply this useful framework to evaluate the mediating role of exposure to phthalates and their replacements in the association between race/ethnicity and fetal growth. We had the following objectives: 1) estimate the differences in phthalate replacement biomarkers by race/ethnicity; 2) evaluate the association between race/ethnicity and birthweight z-scores; 3) assess associations between phthalate replacement biomarkers and birthweight z-scores; and 4) examine mediation of the association between race/ethnicity and birthweight z-scores by phthalate exposure biomarkers. To inform our mediation approach, we also assessed exposure-mediator interaction (i.e., between race/ethnicity and phthalate exposure biomarkers) in the relationship with birthweight z-scores.

## Methods

### Study design and population

We used data from a prospective pregnancy cohort designed to examine placental, maternal, and fetal cardiovascular markers as predictors of adverse pregnancy outcomes. Participants 18–50 years of age with a singleton pregnancy were recruited before 14 weeks of gestation at Eastern Virginia Medical School and the University of Texas Medical Branch between 2016 and 2020. Before enrollment and study-related procedures were initiated, informed consent was obtained from each willing participant. Beginning in 2017, participants in the parent study were also invited to participate in the Human Placenta and Phthalates (HPP) study, designed to examine urinary biomarkers of chemical exposures and placental and fetal development.^25^ The parent study enrolled 610 participants; 303 (49.7%) were also participants in the HPP study, of which 1,866 urine samples among 303 participants were collected, corresponding to an average of approximately 6.2 samples per participant. Participants with at least one urine sample were included in prior longitudinal biomarker analyses, and no minimum threshold of three samples was required for inclusion in the present pregnancy-averaged exposure analysis. The HPP study included up to eight study visits per participant with urine sample collection, ultrasounds, and questionnaires.^25^ The first four visits occurred approximately every 2 weeks at a mean of 12, 14, 17, and 20-weeks gestation. The following four visits occurred every 4 weeks at a mean of 24, 28, 32, and 36-weeks gestation. Participants with placentas in an abnormal position, fetal or umbilical cord abnormalities, and prisoners were excluded from the study.

Maternal medical history and pregnancy outcomes were ascertained from medical records. Demographics and behavioral characteristics, including smoking, drug use, alcohol use, and medications, were collected via questionnaire. All medical data were manually reviewed by study staff for quality control purposes. This study was approved by the Institutional Review Boards of each participating institution. Analysis of deidentified urine samples by the Centers for Disease Control and Prevention (CDC) laboratory was determined not to constitute engagement in human subject’s research.

### Race and ethnicity

Participant race and ethnicity were self-reported in two separate questions, and responses were categorized as NHW, NHB, Hispanic, or “Other” participants, which included groups with small sample sizes non-Hispanic (Asian, Native Hawaiian, or Pacific Islander, and Multiracial). Race/ethnicity information was provided by self-report. In this analysis, we examine race/ethnicity to represent the unmeasured social factors that drive race/ethnicity associations with both exposure to phthalates and replacements (e.g., targeted marketing of personal care and consumer products and socioeconomic stressors),^4^ as well as birthweight.

### Phthalate and replacement biomarker assessment

Phthalate and replacement metabolite concentrations were quantified in urine. At each study visit, total urine voids were collected from each participant in sterile polypropylene containers and shipped in batches to a central processing laboratory in Durham, North Carolina. Samples were stored at −80 °C until they were shipped frozen overnight to the National Center for Environmental Health at CDC for analysis. Further details on the analytic process have been described previously.^25,26^ Briefly, phthalate and replacement metabolite quantification involved enzymatic hydrolysis of the metabolites from their conjugated form, automated online solid-phase extraction, and separation with liquid chromatography tandem mass spectrometry. The following metabolites were quantified: monoethyl phthalate (MEP), monobenzyl phthalate, mono-3-carboxy propyl phthalate, monocarboxy-isononyl phthalate, mono-n-butyl phthalate (MBP), monohydroxybutyl phthalate, mono-isobutyl phthalate (MiBP), monohydroxy-isobutyl phthalate, mono-2-ethylhexyl phthalate, mono-2-ethyl-5-hydroxyhexyl phthalate, mono-2-ethyl-5-oxohexyl phthalate, mono-2-ethyl-5-carboxypentyl phthalate (MECPP), monooxononyl phthalate, monocarboxyisooctyl phthalate (MCOP), mono-2-ethyl-5-hydroxyhexyl terephthalate, and mono-2-ethyl-5-carboxypentyl terephthalate (MECPTP). Replacement metabolites included: cyclohexane-1,2-dicarboxylic acid monohydroxyisononyl ester (MHiNCH), and cyclohexane-1,2-dicarboxylic acid monocarboxyisooctyl ester (MCOCH).

Concentrations below the limit of detection were imputed as limit of detection/√2.^27^ Molar sums were created by summing concentrations (nmol/ml) of the metabolites from the same parent compound, including: di-n-butyl phthalate (ΣDnBP) from MBP and monohydroxybutyl phthalate; di-iso-butyl phthalate (ΣDiBP) from MiBP and monohydroxy-isobutyl phthalate; di-2-ethylhexyl phthalate (ΣDEHP) from mono-2-ethylhexyl phthalate, mono-2-ethyl-5-hydroxyhexyl phthalate, mono-2-ethyl-5-oxohexyl phthalate, and MECPP; di-isononyl phthalate (ΣDiNP) from monooxononyl phthalate and MCOP; di-2-ethylhexyl terephthalate (ΣDEHTP) from mono-2-ethyl-5-hydroxyhexyl terephthalate and MECPTP; and 1,2-cyclohexane dicarboxylic acid, diisononyl ester replacement biomarkers (ΣDINCH) from MHiNCH and MCOCH.^28^ ΣDnBP, ΣDiBP, ΣDEHP, ΣDiNP, ΣDEHTP, and ΣDINCH were converted to ng/ml by multiplying by the molecular weights of MBP, MiBP, MECPP, MCOP, MECPTP, and MHiNCH, respectively. All individual metabolites and summed metabolites are termed “phthalate and replacement biomarkers” for this analysis.

Biomarker concentrations were subsequently adjusted for urine dilution using the following formula: *P*_*c*_ = *P* [(1.019–1)/(SG−1)], where *P*_*c*_ was the specific gravity (SG)-corrected biomarker concentration, *P* was the measured metabolite concentration (ng/ml), 1.019 was the median specific gravity from all urine samples collected in pregnancy, and SG was the SG of each urine sample.^29^

The SG-corrected geometric means of all available phthalate or replacement biomarker measurements in pregnancy were calculated as a measure of the average phthalate or replacement exposure biomarker across pregnancy. To improve the comparability of the estimates across all biomarkers, each biomarker concentration was then natural log-transformed and interquartile range standardized in subsequent analyses.

### Birthweight z-scores

We abstracted birthweight (grams), gestational age at delivery (weeks), and infant sex (male, female) from the medical records. For our primary outcome measure, we calculated birthweight z-scores using a U.S. population-based reference that standardizes birthweight for gestational age at delivery and infant sex, but not maternal race or ethnicity.^30^ We used a nonrace/ethnicity-specific reference because race- and ethnicity-specific fetal growth standards may normalize differences in birthweight that reflect structural and social inequities rather than biological variation.

### Covariates

The following study covariates were ascertained at the first study visit: maternal age; prepregnancy body mass index (BMI, kg/m^2^); highest education level (high school or equivalent, some college, 4-year college degree); parity (0 vs. 1 or more); smoking, alcohol, and drug use in the 3 months before pregnancy; fetal sex; and study site. These variables were identified as possible confounders of the association between phthalate exposure biomarkers and birthweight z-scores based on our previous work.^25^ We furthermore included these covariates in models of race/ethnicity and birthweight z-scores, as well as race/ethnicity and phthalate exposure biomarkers, because these factors are strong predictors of fetal growth as well as phthalate exposure.^15,31,32^ Including these adjustments allows for a more accurate assessment of phthalates’ independent effects in the relationship between racial and ethnic disparities and fetal growth.

### Statistical analysis

Statistical analyses were performed using R version 4.3.1 (Vienna, Austria). Distributions of maternal characteristics were examined (means ± standard deviation [SD] and n [%]). We also examined the distributions of pregnancy covariates by participant race/ethnicity.

We performed three initial tests to examine relationships between race/ethnicity, phthalate exposure biomarkers, and birthweight z-scores, all using generalized linear regression models. First, we estimated the difference in individual phthalate exposure biomarkers by race/ethnicity. Second, we estimated the associations between race/ethnicity and birthweight z-scores. Third, we estimated the associations between phthalate exposure biomarkers and birthweight z-scores. As above, all models were adjusted for the same covariates (maternal age, prepregnancy BMI, highest education level, parity, smoking, alcohol, drug use, fetal sex, and study site). A *P*-value cutoff of <0.10 was used as a threshold to identify phthalate exposure biomarkers that were associated with race/ethnicity and birthweight z-scores that should be examined in subsequent mediation analyses.

We also evaluated an exposure-mediator interaction (i.e., whether phthalate exposure biomarkers modify the association between race/ethnicity and birthweight z-score) to inform our mediation models. We also used a less conservative threshold of a *P*-value cutoff of <0.10 to identify candidate biomarkers for exploratory mediation analyses because mediation can be difficult to detect in smaller sample sizes, particularly when the exposure, mediator, and outcome pathways may each have small effects. This threshold was used only for mediator screening and not as a criterion for definitive statistical significance.

We used the mediation package in R to perform causal inference-based mediation analysis to investigate whether phthalate exposure biomarkers mediate the relationship between race/ethnicity and birthweight z-scores. This method separates the effect of race/ethnicity on birthweight z-scores into three components: 1) the total effect of race/ethnicity on birthweight z-scores; 2) the effect of race/ethnicity on birthweight z-scores mediated through each individual phthalate exposure biomarker (indirect effect); and 3) the effect of race/ethnicity on birthweight z-scores not mediated through the phthalate exposure biomarkers (direct effect).^33^ The fraction of the total effect mediated by each phthalate exposure biomarker was expressed as a percentage (i.e., percent mediated).

## Results

### Participant characteristics

Maternal and pregnancy characteristics for the overall study population are presented in Table 1. Among 303 participants enrolled in the study, we excluded five participants from this analysis who identified as non-Hispanic (Asian, Hawaiian/Pacific Islander, or Multiracial) due to the small sample sizes in these groups and restricted the sample size to complete cases only (analytic sample = 271). Participant characteristics were similar between the enrolled and the analytic sample, and those who were excluded (n = 32) (Supplemental Table 5; https://links.lww.com/EE/A445). Previously published descriptions of participant characteristics for the parent Human Placenta cohort and analytic subsets suggest that participants included in biomarker analyses were generally similar to the parent cohort with respect to key sociodemographic and clinical characteristics.^25,34^ On average, participants were 26 years of age with a prepregnancy BMI of 25 kg/m^2^. Most (43%) participants self-reported as NHB, and 43% had a high school or equivalent educational level. On average, participants delivered at a gestational age of 38 weeks with an infant weighing 3,135*g*. Phthalates and replacement metabolites were widely detected, with 12 metabolites detected in over 90% of the samples, as described in our previous work.^25,34^

**Table 1.** Participant characteristics in the Human Placenta and Phthalates study, 2017–2020

	Mean ± SD (n) or n (%)	
Characteristics	Enrolled (n = 303)	Analytic^a^ (n = 271)
Maternal age, years	26.5 ± 5.2	26.4 ± 5.2
Missing	2 (0.7)	0 (0.0%)
Maternal BMI, kg/m^2^	24.9 ± 4.3	24.8 ± 4.3
Missing	5 (1.7)	0 (0.0%)
Study site		
EVMS	218 (72.0%)	194 (72.0%)
UTMB	85 (28.1%)	76 (28.0%)
Any previous pregnancies	222 (73.3%)	202 (74.5%)
Self-identified race and ethnicity		
Non-Hispanic white	118 (38.9%)	107 (39.5%)
Non-Hispanic black	131 (43.2%)	120 (44.3%)
Hispanic	49 (16.2%)	44 (16.2%)
Other^b^	5 (1.7%)	0 (0%)
Highest education level		
High school or equivalent	131 (43.2%)	124 (45.8%)
Some college	123 (40.6%)	113 (41.7%)
4-year college degree	39 (12.9%)	34 (12.5%)
Missing	10 (3.3%)	0 (0.0%)
Smoked in 3 months before pregnancy	97 (32.0%)	90 (33.2%)
Missing	2 (0.7%)	0 (0.0%)
Alcohol use in 3 months before pregnancy	31 (10.2%)	27 (10.0%)
Missing	1 (0.3%)	0 (0.0%)
Drug use in 3 months before pregnancy	39 (12.9%)	37 (13.7%)
Missing	1 (0.3%)	0 (0.0%)
Gestational age at delivery, weeks	38.1 ± 2.0	38.1 ± 1.7
Birthweight, grams	3,135 ± 557	3,146 ± 525.6
Infant sex		
Male	147 (48.5%)	137 (50.6%)
Female	144 (47.5%)	133 (49.1%)
Missing	12 (4.0%)	0 (0.0%)

a Analytic sample was limited to those with no missing data and was restricted to complete cases, excluding participants with missing data on exposure, outcome, or covariates.

b Other race and ethnicity category includes participants identifying as non-Hispanic (Asian, Hawaiian or Pacific Islander, or Multiracial).

BMI indicates body mass index; EVMS, Eastern Virginia Medical School; SD, standard deviation; UTMB, University of Texas Medical Branch.

In adjusted models, NHB participants had 0.43 lower birthweight z-scores (95% CI = −0.67, −0.19) compared with NHW participants (Table 2). Birthweight z-scores for Hispanic participants were the same as those observed for NHW participants (B = −0.01, 95% CI = −0.34, 0.33).

**Table 2.** Adjusted^a^ differences in birthweight-for-gestational-age z-scores (95% confidence intervals) in non-Hispanic black and Hispanic participants compared with non-Hispanic white participants

	Birthweight-for-gestational-age z-score	
	Adjusted models	
Race/ethnicity	Difference	95% CI
Non-Hispanic white	Ref.	Ref.
Non-Hispanic black	−0.43	−0.67, −0.19
Hispanic	−0.01	−0.34, 0.33

a Adjusted models included maternal age, prepregnancy body mass index, highest education level, parity, smoking, alcohol, drug use, fetal sex, and study site.

CI indicates confidence interval; Ref, reference group; SD, standard deviation.

**Figure 1.**
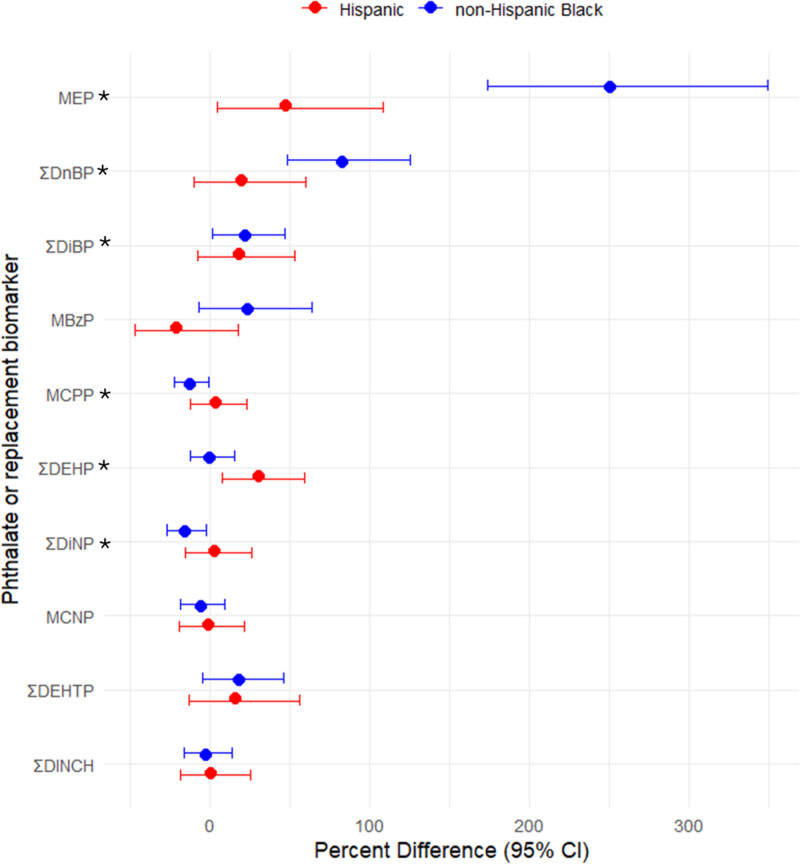
Adjusted^a^ percent differences (95% confidence intervals) in gestational phthalate and replacement biomarker urinary concentrations for Hispanic and non-Hispanic black participants compared with non-Hispanic white participants. ^a^Adjusted for maternal age, prepregnancy body mass index, highest education level, parity, smoking, alcohol, and drug use, fetal sex, and study site. *Associations for MEP, ΣDnBP, ΣDiBP, MCPP, ΣDEHP, ΣDiNP were statistically significant (*P* < 0.05). ΣDEHP indicates sum of di-2-ethylhexyl phthalate metabolites; ∑DEHTP, sum of di-2-ethylhexyl terephthalate metabolites; ∑DiBP, sum of di-iso-butyl phthalate metabolites; ∑DINCH, sum of 1,2-cyclohexane dicarboxylic acid, diisononyl ester metabolites; ∑DiNP, sum of di-isononyl phthalate metabolites; ∑DnBP, Di-n-butyl phthalate; CI, confidence interval; MBzP, monobenzyl phthalate; MCNP, monocarboxyisononyl phthalate; MCPP, mono-3-carboxypropyl phthalate; MEP, monoethyl phthalate.

NHB participants exhibited higher urinary concentrations of most biomarkers compared with NHW participants after adjustment for covariates (Figure 1, Supplemental Table 1; https://links.lww.com/EE/A445). For example, MEP, ΣDnBP, and ΣDiBP concentrations were 250.0% higher (95% CI = 173.0, 349.0), 83% higher (95% CI = 48.5, 125.4), and 22.1% higher (95% CI = 1.6, 46.8), respectively, in NHB compared with NHW participants. For Hispanic participants, phthalate and replacement biomarker concentrations were more like those observed among NHW participants. Yet, MEP and ΣDEHP concentrations were 47.3% higher (95% CI = 4.4, 108.0) and 30.8% higher (95% CI = 7.7, 58.9), respectively, in Hispanic participants compared with NHW participants.

**Figure 2.**
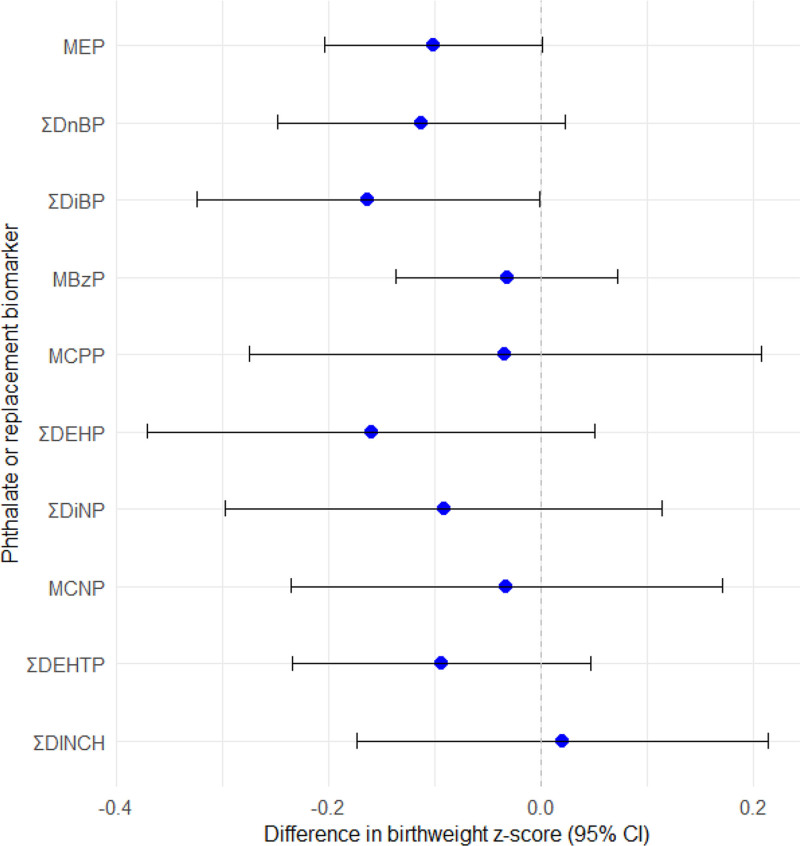
Adjusted^a^ differences (95% confidence intervals) in birthweight-for-gestational-age z-score for an interquartile range difference in pregnancy-averaged gestational phthalate and replacement biomarker urinary concentrations. ^a^Adjusted for maternal age, prepregnancy body mass index, highest education level, parity, smoking, alcohol, drug use, fetal sex, and study site. ∑DEHP indicates sum of di-2-ethylhexyl phthalate; ∑DEHTP, sum of di-2-ethylhexyl terephthalate metabolites; ∑DiBP, sum of di-iso-butyl phthalate metabolites; ∑DINCH, sum of 1,2-cyclohexane dicarboxylic acid, diisononyl ester metabolites; ∑DiNP, sum of di-isononyl phthalate metabolites; ∑DnBP, sum of di-n-butyl phthalate metabolites; CI, confidence interval; MBzP, monobenzyl phthalate; MCNP, monocarboxyisononyl phthalate; MCPP, mono-3-carboxypropyl phthalate; MEP, monoethyl phthalate.

Finally, we observed that several phthalate and replacement biomarkers were inversely associated with birthweight z-scores (Figure 2, Supplemental Table 2; https://links.lww.com/EE/A445). For example, an interquartile range difference in pregnancy-average MEP was associated with 0.10 lower birthweight z-scores (95% CI = −0.20, 0.00), and ΣDiBP was associated with −0.16 lower birthweight z-scores (95% CI = −0.32, −0.002). We also noted that ΣDnBP was marginally associated with lower birthweight z-scores (B = −0.11, 95% CI = −0.25, 0.02).

**Figure 3.**
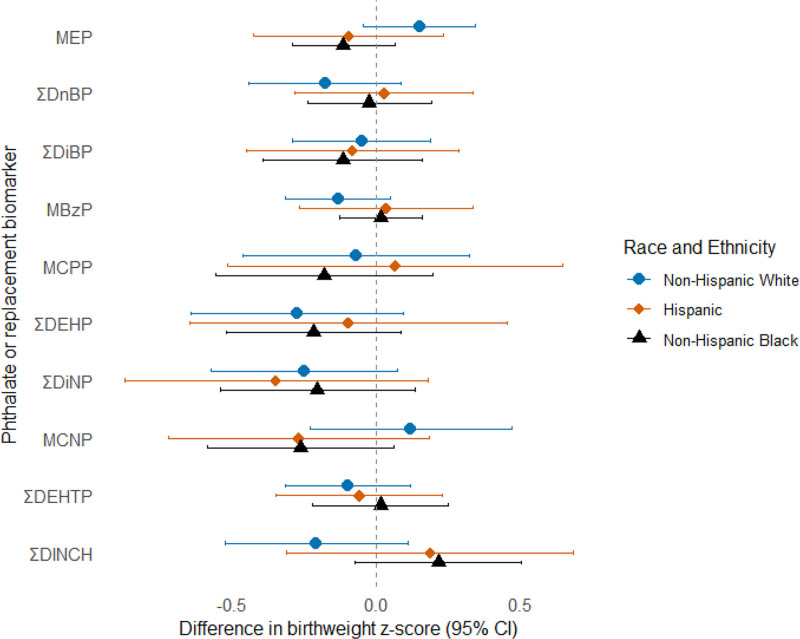
Adjusted^a^ differences (95% confidence intervals) in birthweight-for-gestational-age z-score for an interquartile range difference in pregnancy-averaged gestational phthalate and replacement biomarkers in models stratified by participant race/ethnicity.^a^Adjusted interquartile percent differences in exposure biomarkers adjusted for maternal age, body mass index, highest education level, parity, smoking, alcohol, drug use, fetal sex, and study site. None of the associations shown were statistically significant (*P* > 0.05). ∑DEHP indicates sum of di-2-ethylhexyl phthalate metabolites; ∑DEHTP, sum of di-2-ethylhexyl terephthalate metabolites; ∑DiBP, sum of di-iso-butyl phthalate metabolites; ∑DINCH, sum of 1,2-Cyclohexane dicarboxylic acid, diisononyl ester metabolites; ∑DiNP, sum of di-isononyl phthalate metabolites; ∑DnBP, sum of di-n-butyl phthalate metabolites; CI, confidence interval; MBzP, monobenzyl phthalate; MCNP, monocarboxyisononyl phthalate; MCPP, mono-3-carboxypropyl phthalate; MEP, monoethyl phthalate.

We did not observe significant (*P* < 0.10) interactions between gestational phthalate and replacement biomarkers and race/ethnicity for the associations with birthweight z-scores (Figure 3, Supplemental Table 3; https://links.lww.com/EE/A445). However, we noted that MEP and monocarboxy-isononyl phthalate had inverse associations with birthweight z-scores among NHB and Hispanic participants, and positive associations among NHW participants. ΣDINCH showed inverse associations with birthweight z-scores among NHW participants and positive associations with birthweight z-scores among NHB and Hispanic participants. Because these associations were not significant, we did not include exposure-mediator interaction terms in our mediation models.

**Figure 4.**
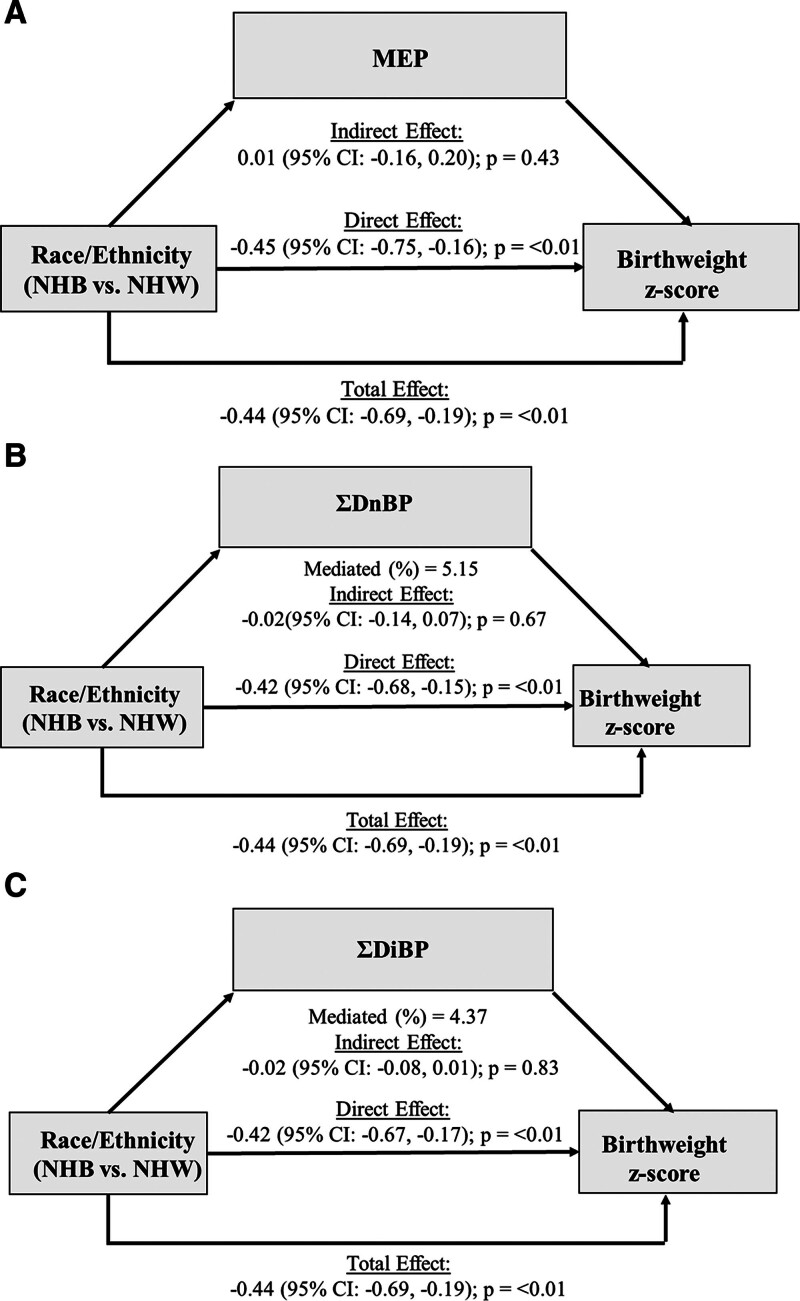
Mediation framework and adjusted indirect, direct, and total effect estimates for associations between participant race and ethnicity (non-Hispanic Black [NHB] vs. non-Hispanic White [NHW]) and birthweight-for-gestational-age z-score for (A) monoethyl phthalate (MEP), (B) the sum of di-n-butyl phthalate metabolites (ΣDnBP), and (C) the sum of di-iso-butyl phthalate metabolites (ΣDiBP). Models were restricted to NHB and NHW participants (n = 227) because no differences in birthweight-for-gestational-age z-scores were observed for Hispanic participants compared with NHW participants. Percent mediated was not calculated for MEP because the indirect and total effect estimates were in opposite directions. Models were adjusted for maternal age, prepregnancy body mass index, highest education level, parity, smoking, alcohol use, drug use, fetal sex, and study site. CI, confidence interval.

Displayed in (Figure 4 and Supplemental Table 4; https://links.lww.com/EE/A445) are the results of the causal mediation analysis evaluating phthalate and replacement biomarkers as mediators of the association between race/ethnicity and birthweight z-scores. We performed mediation analyses for MEP, ΣDiBP, and ΣDnBP as they were associated with both race/ethnicity and birthweight z-scores based on a *P*-value cutoff of <0.10, and we only included NHB and NHW participants as they exhibited differences in birthweight z-scores. We observed a significant total effect of race/ethnicity on birthweight z-scores and a direct effect of race/ethnicity on birthweight z-scores, as expected. However, the indirect effects of the association between race/ethnicity and birthweight z-scores by MEP, ΣDiBP, or ΣDnBP were all nonsignificant. Furthermore, the percent mediated was quite low for ΣDiBP (4.4%) and ΣDnBP (5.2%). Given that the indirect and total effect estimates for MEP were in opposing directions, we did not report the percent mediated for that model.

## Discussion

The purpose of this study was to examine the mediating role of exposure to phthalates and their replacements in the association between race/ethnicity and fetal growth. We first observed differences in phthalate exposure biomarkers by race/ethnicity. These findings generally suggested that NHB, and to a lesser extent, Hispanic participants, exhibited heightened phthalate biomarker concentrations during pregnancy relative to NHW participants. Second, we observed associations between race/ethnicity and birthweight z-scores. In line with prior research,^25,35^ these findings suggested that NHB participants had smaller infants relative to NHW participants. Third, some phthalate exposure biomarkers (MEP, DnBP, DiBP) were associated with lower birthweight z-scores. Yet, we observed only modest, nonsignificant mediation of the association between race/ethnicity and birthweight z-scores by phthalate exposure biomarkers. While this was contrary to our hypotheses, these findings suggest that the well-known differences in birthweight in NHB compared with NHW mothers may be attributable to factors other than phthalate exposure, despite the fact that exposures appear to be so different in these two groups.

There is strong support for the hypothesis that phthalate exposure may contribute to the known racial differences in birthweight.^6,36,37^ Consistent with prior research,^15,20^ our study observed differences in phthalate exposure by race/ethnicity; for example, NHB individuals exhibited higher urinary concentrations of ∑DnBP compared with NHW individuals. This is consistent with previous work from our study, where we showed that longitudinal profiles of phthalate metabolites, which clustered individuals into low, medium, or high exposure groups, showed that NHB participants were more likely to be in the high exposure group compared with participants from other races/ethnicities.^34^ These findings align with a growing body of literature suggesting that racial and ethnic differences in phthalate exposure are socially patterned and structurally produced, reflecting differences in product marketing, product availability, occupational and housing-related exposures, diet and food packaging, neighborhood retail environments, and broader structural determinants that shape exposure opportunities.^18^ In particular, products such as vaginal douches, feminine sprays, powders, and wipes are often marketed toward women of color and frequently contain phthalates or other EDCs, contributing to higher exposure burdens in some racial and ethnic groups.^17^

In addition to personal care product use, differences in exposure to phthalates by race/ethnicity may exist based on residential factors. Communities of color are more likely to reside in areas with higher levels of environmental pollution (e.g., industrial waste) and have less access to fresh foods, leading to increased consumption of processed and packaged products, another potential source of phthalate exposure.^20,24^

At the same time, there is extensive mechanistic research linking phthalate exposure to lower birthweight.^25,38–40^ Phthalates can cross the placental and blood-brain barriers^41^ and may interfere with normal fetal development through endocrine disruption,^42,43^ oxidative stress, inflammation, or downstream effects on placental function.^44^

Thus, scientific evidence provides solid support for the hypothesis that exposure to phthalates may mediate, at least in part, the known associations between race/ethnicity and birthweight. Examining environmental exposures as mediators in the relationship between race/ethnicity and adverse health outcomes is a concept proposed by James-Todd and colleagues.^20,24^ We chose to explore this modeling approach in the current study because we observed the combination of overlapping exposure-mediator and mediator-outcome associations in our dataset. However, several other approaches have been utilized in the literature to examine how race/ethnicity, birthweight, and chemical exposures are interrelated. Ward et al. proposed another valuable conceptual framework of effect modification, suggesting that racial differences may arise from differences in exposures, differences in vulnerability to exposures, or both.^45^ We illustrated the possible impact of differences in exposure by race/ethnicity in a separate study where a hypothetical reduction in phthalate exposure among Black participants to mirror the distribution in white participants resulted in a decrease in rates of preterm birth.^46^ Regarding differences in vulnerability to exposures, the health effects of phthalates may be compounded by psychosocial stressors that are disproportionately experienced by people of color.^47^ For example, in Ferguson et al., we observed that associations between phthalates and replacement biomarkers and preterm birth were greatest in magnitude among study participants who experienced stressful life events during pregnancy.^48^ While it was beyond the scope of our current project to explore associations under each of these different frameworks, we did examine effect modification of phthalate-birthweight associations by participant race/ethnicity to determine whether interactions should be included in our mediation models. However, we did not observe evidence of effect modification in the associations.

### Limitations

This study had some limitations. First, although we adjusted for known covariates, we cannot rule out residual confounding from factors such as physical activity and diet, which are known to be associated with race/ethnicity, phthalate exposure, and birthweight. At the same time, our previous work found that adjusting for these variables did not materially change the effect estimates in the relationship between phthalates and fetal growth.^49^ Second, given that race is a social construct, not a biological variable, the associations between race/ethnicity and fetal growth likely reflect social, environmental, and structural factors. Because these factors were not fully captured in our data, it limits the interpretability of race-based associations and underscores the need for more nuanced measures of lived experiences. Due to sample size constraints, mediation estimates were small and imprecise, which may have reduced our ability to detect modest indirect effects. We were also limited to comparisons between NHB and NHW participants and were unable to assess mediating effects of phthalates in other racial/ethnic groups, which had small sample sizes, which may be of interest in future studies. Lastly, we recognize that phthalate exposures occur as a mixture, and it may be of interest to examine a joint indirect effect. Methods to perform mediation with mixtures are emerging, and this may be important to consider in future work where there is evidence of indirect effects of single chemicals.

### Strengths

Despite these limitations, our study had several important strengths. Notably, we applied causal mediation analysis to evaluate the indirect effects of environmental exposures on racial/ethnic differences in birth outcomes, a methodology that remains underutilized in this field.^21–23,33^ We also employed a rigorous exposure assessment strategy, using repeated biomarker measurements from up to eight time points per participant across pregnancy to calculate stable, within-subject gestational averages of phthalate and replacement biomarker concentrations. Furthermore, our racially diverse cohort with roughly equal representation of NHB and NHW participants provided a strong basis for examining mediators that could drive differences in birthweight observed in these two groups.

## Conclusion

In this well-characterized and racially diverse pregnancy cohort, we observed significant racial/ethnic differences in phthalate biomarker concentrations, associations between race/ethnicity and birthweight z-scores, and associations between specific phthalate exposure biomarkers and fetal growth. Although we did not observe mediation of the race/ethnicity-birthweight association by phthalate and replacement biomarkers in this study, the mediation approach presented may be worth considering to better understand the role of environmental exposures in the relationships between race/ethnicity and adverse birth outcomes when exposure-mediator, mediator-outcome, and exposure-outcome associations are observed. Such an approach may aid in identifying other environmental intermediates that could be targeted in interventions aimed at reducing racial and ethnic differences in adverse birth outcomes.

## Conflicts of interest statement

The authors declare that they have no conflicts of interest with regard to the content of this report.

## Supplementary Material
