# Supplementary material for: Evaluating the mediating role of gestational phthalate and replacement biomarkers in the associations between race/ethnicity and birthweight
Source: Environ Epidemiol. 2026 Aug 7;10(4):e506. doi: 10.1097/EE9.0000000000000506 (PMC13456755; doi:10.1097/EE9.0000000000000506)
Supplement: Supplementary file 1 [file ee9-10-e506-s001.pdf]

Supplemental Table 1. Adjusted[a] percent differences (95% confidence intervals) in gestational urinary concentrations of phthalate and replacement biomarkers for non-Hispanic Black and Hispanic participants compared to non-Hispanic White participants

	Adjusted Models			
	Non-Hispanic Black Participants		Hispanic Participants	
	% Difference	95% CI	% Difference	95% CI
Phthalates				
MEP	250.3	173.3, 349.0	47.3	4.4, 108.0
ΣDnBP	83.0	48.5, 125.4	19.5	-10.5, 59.7
ΣDiBP	22.1	1.6, 46.8	18.3	-8.3, 52.7
MBzP	23.3	-7.3, 63.9	-21.1	-46.9, 17.2
MCPP	-12.7	-22.8, -1.2	3.5	-12.9, 22.8
ΣDEHP	0.1	-12.9, 15.2	30.8	7.7, 58.9
ΣDiNP	-15.9	-27.2, -2.9	3.0	-15.7, 25.9
MCNP	-5.9	-18.9, 9.1	-1.0	-19.4, 21.6
Replacements				
ΣDEHTP	18.1	-4.5, 46.1	16.2	-13.5, 56.2
ΣDINCH	-2.7	-16.6, 13.6	0.8	-18.7, 25.0

Abbreviations: Confidence interval (CI); Monoethyl phthalate (MEP); Sum of di-n-butyl phthalate metabolites (∑DnBP); Sum of di-iso-butyl phthalate metabolites (∑DiBP); Monobenzyl phthalate (MBzP); Mono-3-carboxypropyl phthalate (MCPP); Sum of di-2-ethylhexyl phthalate metabolites (∑DEHP); Sum of di-isononyl phthalate metabolites (∑DiNP); Monocarboxyisononyl phthalate (MCNP); Sum of di-2-ethylhexyl terephthalate metabolites (∑DEHTP); Sum of 1,2-Cyclohexane dicarboxylic acid, diisononyl ester metabolites (∑DINCH).
Results presented are the percent difference in gestational phthalate and replacement biomarker concentrations (ng/mL) for comparisons to non-Hispanic White participants from linear regression models.
[a]Adjusted models included adjustment for maternal age, body mass index, highest education level, parity, smoking, alcohol, drug use, fetal sex, and study site.

Supplemental Table 2. Adjusted[a] differences and 95% confidence intervals for associations of pregnancy-averaged urinary concentrations of gestational phthalate and replacement biomarkers with birthweight-for-gestational-age z-score

	Birthweight-for-gestational-age z-score		
	Adjusted Models		
	Difference	95% CI	p-value
Phthalates			
MEP	-0.10	-0.20, 0.00	0.05
ΣDnBP	-0.11	-0.25, 0.02	0.10
ΣDiBP	-0.16	-0.32, -0.00	0.04
MBzP	-0.03	-0.14, 0.07	0.54
MCPP	-0.03	-0.28, 0.21	0.78
ΣDEHP	-0.16	-0.37, 0.05	0.13
ΣDiNP	-0.09	-0.29, 0.11	0.38
MCNP	-0.03	-0.23, 0.17	0.75
Replacements			
ΣDEHTP	-0.09	-0.23, 0.05	0.19
ΣDINCH	0.02	-0.17, 0.21	0.84

Abbreviations: Confidence interval (CI);Monoethyl phthalate (MEP); Sum of di-n-butyl phthalate metabolites (∑DnBP); Sum of di-iso-butyl phthalate metabolites (∑DiBP); Monobenzyl phthalate (MBzP); Mono-3-carboxypropyl phthalate (∑MCPP); Sum of di-2-ethylhexyl phthalate metabolites (∑DEHP); Sum of di-isononyl phthalate metabolites (∑DiNP); Monocarboxyisononyl phthalate (MCNP); Sum of di-2-ethylhexyl terephthalate metabolites (∑DEHTP); Sum of 1,2-Cyclohexane dicarboxylic acid, diisononyl ester metabolites (∑DINCH).

Results presented are the differences for birthweight-for-gestational-age z-score outcomes for a one-unit increase in log-transformed gestational phthalate and replacement biomarkers from linear regression models.

[a]Adjusted models included adjustment for maternal age, body mass index, highest education level, parity, smoking, alcohol, drug use, fetal sex, and study site.

Supplemental Table 3. Adjusted[a] differences and 95% confidence intervals for associations of gestational urinary concentrations of phthalate and replacement biomarkers with birthweight-for-gestational-age z-score by race and ethnicity

	Birthweight-for-gestational-age z-score						
	Non-Hispanic White		Non-Hispanic Black		Hispanic		p-value
	Difference	95% CI	Difference	95% CI	Difference	95% CI	
Phthalates							
MEP	0.15	-0.04, 0.35	-0.11	-0.29, 0.07	-0.01	-0.42, 0.23	0.69
ΣDnBP	-0.18	-0.44, 0.09	-0.02	-0.24, 0.19	0.03	-0.28, 0.34	0.37
ΣDiBP	-0.05	-0.29, 0.19	-0.11	-0.39, 0.16	-0.08	-0.45, 0.29	0.39
MBzP	-0.13	-0.31, 0.05	0.02	-0.13, 0.16	0.04	-0.27, 0.34	0.35
MCPP	-0.07	-0.46, 0.33	-0.18	-0.56, 0.20	0.07	-0.52, 0.65	0.28
ΣDEHP	-0.27	-0.64, 0.01	-0.22	-0.52, 0.09	-0.09	-0.65, 0.46	0.34
ΣDiNP	-0.25	-0.57, 0.08	-0.20	-0.54, 0.13	-0.35	-0.87, 0.18	0.36
MCNP	0.12	-0.23, 0.47	-0.26	-0.58, 0.06	-0.27	-0.72, 0.18	0.23
Replacements							
ΣDEHTP	-0.10	-0.32, 0.12	0.02	-0.22, 0.25	-0.06	-0.35, 0.23	0.30
ΣDINCH	-0.21	-0.52, 0.11	0.22	-0.07, 0.51	0.19	-0.31, 0.69	0.25

Abbreviations: Confidence interval (CI); Monoethyl phthalate (MEP); Sum of di-n-butyl phthalate metabolites (∑DnBP); Sum of di-iso-butyl phthalate metabolites (∑DiBP); Monobenzyl phthalate (MBzP); Mono-3-carboxypropyl phthalate (MCPP); Sum of di-2-ethylhexyl phthalate metabolites (∑DEHP); Sum of di-isononyl phthalate metabolites (∑DiNP); Monocarboxyisononyl phthalate (MCNP); Sum of di-2-ethylhexyl terephthalate metabolites (∑DEHTP); Sum of 1,2-Cyclohexane dicarboxylic acid, diisononyl ester metabolites (∑DINCH).

Results presented are the differences for birthweight-for-gestational-age z-score outcomes for a one-unit increase in log-transformed gestational phthalate and replacement biomarkers from linear regression models.

[a]Models included adjustment for maternal age, body mass index, highest education level, parity, smoking, alcohol, drug use, infant sex, and study site. P-value presents results from the model with an interaction term between race and ethnicity and biomarkers. Race and ethnic-specific results are from models stratified by race and ethnicity of the gestational parent.

Supplemental Table 4. Mediation[a] of the association between participant race and ethnicity (non-Hispanic Black vs. non-Hispanic White) and birthweight-for-gestational-age z-score by pregnancy-average urinary concentrations of phthalate and replacement biomarkers (n = 227)[b]

Phthalates	Direct Effect (95% CI)	Direct Effect p value	Indirect Effect (95% CI)	Indirect p-value	Total Effect (95% CI)	Total p-value	Percent Mediated (%)
MEP	-0.45 (-0.75, -0.16)	0.003	0.01 (-0.16, 0.20)	0.43	-0.44 (-0.69, -0.19)	<0.001	--
ΣDnBP	-0.42 (-0.68, -0.15)	<0.001	-0.02(-0.14, 0.07)	0.67	-0.44 (-0.69, -0.19)	<0.001	5.15
ΣDiBP	-0.42 (-0.67, -0.17)	<0.001	-0.02 (-0.08, 0.01)	0.83	-0.44 (-0.69, -0.19)	<0.001	4.37

Abbreviation: Confidence interval (CI); Monoethyl phthalate (MEP); Sum of di-n-butyl phthalate metabolites (ΣDnBP)); Sum of di-iso-butyl phthalate metabolites (ΣDiBP). [a]Causal mediation analysis using linear regression model estimating direct and indirect effects of race/ethnicity on birthweight-for-gestational-age z-score through each phthalate biomarker as a mediator variable and adjusted for covariates (maternal age, body mass index, highest education level, parity, smoking, alcohol, drug use, fetal sex, and study site).

[b]Models were restricted to non-Hispanic Black and non-Hispanic White participants (n = 227)

--Percent mediated was not calculated for MEP because the indirect effect and total effect estimates were in opposite directions.

Supplemental Table 5. Participant characteristics in the Human Placenta and Phthalates study, 2017-2020

		Mean ± SD (n) or n (%)		
Characteristics		Enrolled (n=303)	Analytic[a] (n=271)	Excluded[c]
Maternal age, years		26.5 ± 5.2	26.4 ± 5.2	27.9 ± 4.9
	Missing	2 (0.7%)	0 (0.0%)	0 (0.0%)
Maternal BMI, kg/m2		24.9 ± 4.3	24.8 ± 4.3	25.3 ± 3.9
	Missing	5 (1.7%)	0 (0.0%)	0 (0.0%)
Study site				
EVMS		218 (72.0%)	194 (72.0%)	23 (71.9%)
UTMB		85 (28.1%)	76 (28.0%)	9 (28.1%)
Any previous pregnancies		222 (73.3%)	202 (74.5%)	20 (62.5%)
Self-identified race and ethnicity				
Non-Hispanic White		118 (38.9%)	107 (39.5%)	11 (34.4%)
Non-Hispanic Black		131 (43.2%)	120 (44.3%)	11 (34.4%)
Hispanic		49 (16.2%)	44 (16.2%)	5 (15.6%)
Other[b]		5 (1.7%)	0 (0%)	5 (15.6%)
Highest education level				
High school or equivalent		131 (43.2%)	124 (45.8%)	7 (21.9%)
Some college		123 (40.6%)	113 (41.7%)	10 (31.2%)
4-year college degree		39 (12.9%)	34 (12.5%)	5 (15.6%)
	Missing	10 (3.3%)	0 (0.0%)	10 (31.2%)
Smoked in 3 months prior to pregnancy		97 (32.0%)	90 (33.2%)	7 (21.9%)
	Missing	2 (0.7%)	0 (0.0%)	2 (6.2%)
Alcohol use in 3 months prior to pregnancy		31 (10.2%)	27 (10.0%)	4 (12.5%)
	Missing	1 (0.3%)	0 (0.0%)	1 (3.1%)
Drug use in 3 months prior to pregnancy		39 (12.9%)	37 (13.7%)	2 (6.2%)
	Missing	1 (0.3%)	0 (0.0%)	1 (3.1%)
Gestational age at delivery, weeks		38.1 ± 2.0	38.1 ± 1.7	37.4 ± 4.4
Birthweight, grams		3135 ± 557	3146 ± 525.6	3137 ± 633.7
Infant sex				
Male		147 (48.5%)	137 (50.6%)	9 (28.1%)
Female		144 (47.5%)	133 (49.1%)	11 (34.4%)

	Missing	12 (4.0%)	0 (0.0%)	12 (37.5%)

Abbreviations: Standard deviation (SD); Body mass index (BMI); University of Texas Medical Branch (UTMB); Eastern Virginia Medical School (EVMS).

[a]*Analytic sample was limited to those with no missing data and were restricted to complete cases, excluding participants with missing data on exposure, outcome, or covariates.*

[b]*Other race and ethnicity category include participants identifying as non-Hispanic(Asian, Hawaiian or Pacific Islander, or Multiracial).*

[c]*Excluded participants with missing data on exposure, outcome, or covariates.*
